# Sequential treatment with bendamustine, obinutuzumab (GA101) and Ibrutinib in chronic lymphocytic leukemia (CLL): final results of the CLL2-BIG trial

**DOI:** 10.1038/s41375-022-01629-7

**Published:** 2022-06-25

**Authors:** Julia von Tresckow, Paula Cramer, Sandra Robrecht, Petra Langerbeins, Anna-Maria Fink, Othman Al-Sawaf, Moritz Fürstenau, Thomas Illmer, Holger Klaproth, Eugen Tausch, Matthias Ritgen, Kirsten Fischer, Clemens-Martin Wendtner, Karl-Anton Kreuzer, Stephan Stilgenbauer, Sebastian Böttcher, Barbara F. Eichhorst, Michael Hallek

**Affiliations:** 1 Department I of Internal Medicine and Center of Integrated Oncology Aachen, Bonn, Cologne, Düsseldorf, German CLL Study Group, Faculty of Medicine and University Hospital of Cologne, University of Cologne, Cologne, Germany; 2 Clinic for Hematology and Stem Cell Transplantation, West German Cancer Center, University Hospital Essen, University of Duisburg-Essen, Essen, Germany; 3 BAG Freiberg-Richter, Jacobasch, Wolf, Illmer, Dresden, Germany; 4 Hämatologische/ Onkologische Praxis Dr. Klaproth, Neunkirchen, Germany; 5 Department of Internal Medicine III, Ulm University, Ulm, Germany; 6 Department of Medicine II, University of Schleswig-Holstein, Kiel, Germany; 7 Department of Hematology, Oncology, Immunology, Palliative Care, Infectious Diseases and Tropical Medicine, Munich Clinic Schwabing, Munich, Germany; 8 Department of Internal Medicine I, Saarland University, Homburg, Germany; 9 Clinic for Internal Medicine III (Hematology, Oncology, Palliative Care), Rostock University Medical School Rostock, Rostock, Germany; 10 Department I of Internal Medicine and Center of Integrated Oncology Cologne-Bonn, CECAD Cluster of Excellence at the University of Cologne, Clinical Research Unit (KFO) 286, German CLL Study Group, University of Cologne, Cologne, Germany

**Keywords:** Phase II trials, Risk factors

## To the Editor:

The prospective, open-label, multicenter phase II trial CLL2-BIG (registered at www.clinicaltrials.gov as # NCT02345863) was the first of the so called BXX trials of the German CLL Study Group (GCLLSG) [1] designed according to the “sequential triple-T” concept of a tailored and targeted treatment aiming at total eradication of minimal residual disease (MRD) [2]. These trials aimed to evaluate novel combination therapies using CD20-antibodies such as obinutuzumab (GA101) and targeted drugs such as ibrutinib with a limited duration of treatment in an all comer population irrespective of firstline (1 L) versus relapse/refractory (RR) therapy, comorbidities and genetic features. Patients with a higher tumor load received two courses of bendamustine as debulking before six cycles of induction therapy (IT) with obinutuzumab and ibrutinib were administered. Patients responding to IT continued with maintenance therapy (MT), consisting of daily ibrutinib and obinutuzumab every three months until achievement of an undetectable MRD (uMRD) remission by flow cytometry (10^−4), confirmed by two consecutive uMRD results in the peripheral blood (PB) within three months, progression, start of new therapy or for up to 24 months, whichever occurred first.

The primary endpoint analysis with an overall response rate of 100% including 47.5% patients with uMRD in PB at the end of IT has been reported previously [3]. Here, we present the final analysis with extended follow-up including the maintenance phase and data on treatment discontinuation triggered by MRD assessment in PB.

61 patients (30 1 L (49.2%), 31 RR (50.8%)) constituted the full analysis set that was defined as all enrolled patients who received at least two complete cycles of IT and used for efficacy analyses according to the study protocol. Safety analyses included all 66 recruited patients who received at least one dose of any compound of the study treatment. Patient demographics are shown in Table 1. After a median observation time of 38.1 months (range 5.4–44.8; 1 L 38.5, RR 37.2) 49 patients (80.3%; 28 1 L, 21 RR) completed the trial as planned. As one RR patient died during IT and another RR patient underwent adverse events (AE) that prohibited further study treatment, 59 of 61 patients (96.7%; 30 1 L, 29 RR) started MT. A median of three maintenance cycles were administered (range 1–8).

**Table 1:** Patient Demographic and Baseline Clinical Characteristics.

Characteristic	FAS	1 L	RR
All patients, *N*	61	30	31
Binet stage, *N* (%)			
C	22 (36.1)	11 (36.7)	11 (35.5)
Age (years)			
Median	66	64.5	67
Range	36–83	36–82	40–83
Total CIRS score			
Median	3	3	2
CLL-IPI Risk Group, *N* (%)			
Low/Intermediate	18 (30.5)	10 (34.5)	8 (16.7)
High	31 (52.5)	15 (51.7)	16 (53.3)
Very high	10 (16.9)	4 (13.8)	6 (20.0)
Missing	2	1	1
IGHV mutational status, *N* (%)			
Unmutated	42 (70.0)	20 (69.0)	22 (71.0)
Mutated	18 (30.0)	9 (31.0)	9 (29.0)
Missing	1	1	0
*TP53* status, *N* (%)			
No aberration	48 (78.7)	26 (86.7)	22 (71.0)
*TP53* mutation and/or *17p* deletion	13 (21.3)	4 (13.3)	9 (29.0)
Response at final restaging			
Overall response rate, *N* (%)	61 (100)	30 (100)	31 (100)
MRD negativity (<10^−4^), *N* (%)	29 (47.5)	16 (53.3)	13 (41.9)

Table **1** shows baseline characteristics as well as genetic risk factors for patients of the full analysis set (FAS), divided into first-line (1 L) and relapsed/refractory (RR) patients, respectively.

*CIRS* cumulative illness rating scale, *IPI* international prognostic index, *IGHV* immunoglobulin heavy-chain variable region.

15 patients (25.4%; 6 1 L, 9 RR) completed 24 months of MT. 11 patients discontinued early: 6 due to AE (10.2%; 2 1 L, 4 RR), 2 each (3.4%) due to PD (2 RR) or refusal of further treatment (2 1 L) and 1 RR due to physician´s decision (1.7%).

33 patients (55.9%; 20 1 L, 13 RR) terminated MT due to uMRD in PB after a median time on MT of 6.0 months (range 3.0–23.3; 1 L: 6.2 months, RR: 5.7 months).

During MT, response was improved in 16 of 59 patients (27.1%) with 6 patients (10.2%) achieving a complete remission (CR) or CR with incomplete recovery of the bone marrow (BM) as best response [4]. 53 patients (89.8%) achieved a partial remission including 32 patients (54.2%) with a clinical CR defined as absence of disease by clinical examination and blood count, but without computed tomography assessment or BM biopsy. 42 of 59 patients (23 1 L, 19 RR) had uMRD in PB at the last staging during MT resulting in an uMRD rate of 71.2% (1 L 76.7%, RR 65.5%). 56.3% of patients without and 76.7% of patients with prior debulking therapy reached uMRD at the last staging during MT.

The median duration of response (DOR) was 38.0 months and the 2-year DOR rate 88.3% (1 L 100.0%, RR 77.4%). The median time to uMRD from the date of enrolment to the date of first uMRD was 10.9 months (1 L 10.2 months, RR 10.9 months) as the first measurement took place after 8 months. The median event free survival (EFS) was 44.8 months with a 3-year EFS rate of 70.9% (1 L 81.8%, RR 60.7%), the median treatment free survival and median time to next treatment were not reached with a 3-year rate of 76.1% (1 L 89.0%, RR 64.0%) and 83.2% (1 L 89.0%, 77.2%), respectively. However, 9 patients (14.8%) received further treatment after the end of the trial (3 1 L (10.0%), 6 RR (19.4%)). Subsequent therapies consisted of chemoimmunotherapy or venetoclax plus obinutuzumab. Five patients (2 1 L, 3 RR) received subsequent therapies with ibrutinib.

**Fig. 1:**
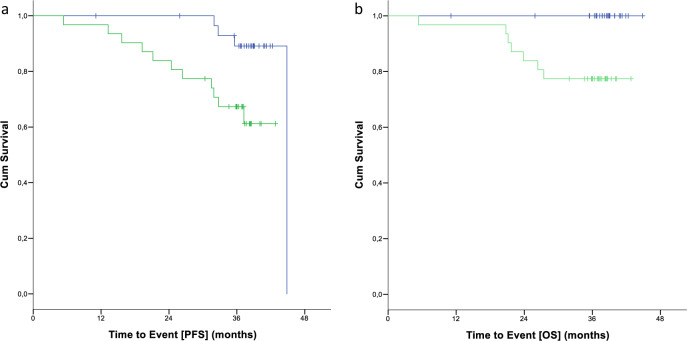
Time to event endpoints. **a** shows the estimated progression free survival (PFS) for patients of the full analysis set (FAS); first line patients are marked in blue, relapsed/refractory patients in green. **b** shows the estimated overall survival (OS) for patients of the full analysis set (FAS); first line patients are marked in blue, relapsed/refractory patients in green.

The estimated median progression free survival (PFS) was 44.8 months with 77.9% (1 L 89.1%, RR 67.3%) being event-free at 3 years (HR 0.230, 95% CI 0.064–0.828; Fig. 1a). Seven of 17 patients without (41.2%) and 8 of 44 patients with prior debulking (18.2%) progressed or died. At 3-years, 57.8% of patients without and 85.7% of patients with debulking were still event-free (HR 0.251, CI 0.084–0.751), In 13 patients with *TP53* aberrations (i.e. *TP53* mutation and/or 17p deletion) 3 (23.1%, 0 1 L, 3 RR) and in 48 patients without genetic *TP53* aberrations 12 events occurred (25%). The PFS rate at 2 years was 76.9% for patients with *TP53* aberrations versus 95.8% for patients without (HR 1.076, 95% CI 0.3–3.86).

In a landmark analysis from last treatment exposure during MT, the median PFS was not reached in patients who stopped MT due to uMRD with a 2-year PFS rate of 82.9%. Five patients with *TP53* aberrations discontinued MT due to uMRD with one progression occuring after 6.2 months after treatment discontinuation. 8 patients with *TP53* aberrations discontinued MT for other reasons than uMRD with two progressions (25.0%).

In another extended PFS landmark analysis of patients with unmutated *IGHV* status who discontinued MT for other reasons, only one progression occurred in 10 patients (10.0%) with prior debulking versus 3 progressions in 8 patients (37.5%) without debulking (HR 0.039, CI 0.003–0.553).

Seven patients died (11.5%) with no deaths occurring in 1 L patients. Causes of deaths included two events of sepsis and one event of pulmonary sepsis, duodenitis, pneumonia and cerebrovascular accident each. One patient died due to PD. No grade 5 AE occurred during MT. Two patients with *TP53* aberrations and very high CLL-IPI died after discontinuation of MT for other reasons than uMRD; one due to AE, one due to PD. One fatality occurred after treatment discontinuation due to uMRD. Overall survival is shown in Fig. 1b.

During MT, 332 CTC grades 1–4 AE were documented. Adjustment of study drugs was performed due to 79 (23.8%) events whereas AE related dose modifications of ibrutinib occurred in 26 patients (44.1%; 15 1 L (50.0%), 11 RR (37.9%)). Most events (85 (25.6%)) were infections or infestations followed by skin and subcutaneous tissue disorders (32 (9.6%)) and gastrointestinal disorders (30 (9.0%)). Most common observed grade 3–4 toxicities during MT were neutropenia (in 11.9% of patients; 1 L 13.3%, RR 10.3%), basal cell carcinoma (in 6.8% of patients; 1 L 13.3%, RR 0), thrombocytopenia (in 5.1% of patients; 1 L 3.3%, RR 6.9%) and pneumonia (in 5.1% of patients; 1 L 6.7%, RR 3.4%). All infections were CTC grade 3 at maximum including one case of fungal pneumonia (CTC grade 3).

17 cases of cardiac disorders were documented, among them 6 cases of atrial fibrillation. 13 bleeding events occurred in 11 of 59 (18.6%) patients.

In conclusion, the CLL2-BIG study demonstrated that sequential therapy with bendamustine, ibrutinib and obinutuzumab showed a very promising efficacy and good safety profile. With the addition of obinutuzmab, no additional toxicity occurred when compared to ibrutinib monotherapy [5] and no increase of bleeding or cardiac events was observed.

By continuation of ibrutinib and obinutuzumab during MT the depth of response could be improved as previously shown for treatment with ibrutinib [5, 6]. Notably, 71.2% of the patients had uMRD in PB at the last staging during MT which is comparable with uMRD rates after venetoclax containing combination regimens [7, 8].

However, even with a longer follow-up this trial will not answer the question whether a fixed-duration treatment is superior to a long-term therapy due to the lack of a randomized, direct comparison. This will be addressed in future trials, e.g. the CLL17 trial (registered at www.clinicaltrials.gov as #NCT04608318).

Nevertheless, even though treatment could be discontinued early, a 3-year PFS rate of 77.9% for the BIG regimen seems comparable with first line treatment with obinutuzumab and ibrutinib as long-term therapy in the ILLUMINATE trial with an estimated 30-month PFS of 79% [9].

Though, our results show that an MRD-guided treatment discontinuation of ibrutinib is promising and feasible for different CLL patient groups including those with unfavorable risk factors. However, it remains to be determined which patients of this heterogeneous study population may have benefited the most.

Prior debulking therapy might be beneficial, possibly due to rapid achievement of uMRD, broad selective pressure or prevention of clonal sweeps caused by prior application of chemotherapy. Whether this is really playing a significant role in overcoming adverse outcomes, especially in patients with unmutated IGHV status or *TP53* aberrations, needs further evaluation.

Therefore, pooled analyses across the BXX trials will be performed. Additionally, a second generation of BXX trials is currently conducted.

Ultimately, these conceptual trials will allow to design more personalized approaches for future CLL therapies.
